# Fast-track surgery versus conventional perioperative management of lung cancer-associated pneumonectomy: a randomized controlled clinical trial

**DOI:** 10.1186/s12957-016-1072-5

**Published:** 2017-01-13

**Authors:** Qing Dong, Kai Zhang, Shouqiang Cao, Jian Cui

**Affiliations:** Department of Thoracic Surgery, the Fourth Affiliated Hospital, Harbin Medical University, 37 Yiyuan Street, Nangang District, Harbin, Heilongjiang 150001 China

**Keywords:** Fast-track surgery, Non-small cell lung cancer, Pneumonectomy, C-reactive protein, Perioperative management

## Abstract

**Background:**

The aim of this study is to investigate the effects of fast-track surgery (FTS) on postoperative recovery, hospital stay, total medical costs, and the complications of pneumonectomy in patients with non-small cell lung cancer (NSCLC).

**Methods:**

Studies were performed between June 2012 and March 2014 in 17 patients received FTS and 18 patients given conventional management (control) after pneumonectomy in the Department of Thoracic Surgery, the Fourth Affiliated Hospital of Harbin Medical University. Patients were evaluated based on their days to achieve the first postoperative flatus, C-reactive protein (CRP) at postoperative day (POD) 1–7, the length of hospital stay, the medical costs, and postoperative complications.

**Results:**

The results showed that in the FTS group, latency to the first postoperative flatus (1.5 ± 0.6 versus 3.1 ± 0.8 s in controls, *P* < 0.0001), CRP (71.36 ± 5.48 versus 80.71 ± 8.32 mg/L in at POD 7, *P* < 0.0001), the length of hospital stay (18.1 ± 1.4 versus 27.4 ± 6.6 days, *P* < 0.0001), and the medical costs (29.9 ± 2.7 versus 37.2 ± 3.6 thousand Chinese Yuan, *P* < 0.0001) were significantly reduced compared to the group receiving conventional management. FTS group also had a relatively lower postoperative complication rate (23.5% of 17 versus 33.3% of 18 in control group) although it was statistically insignificant (*P* = 0.711).

**Conclusions:**

These results indicate that application of the FTS in NSCLC pneumonectomy efficiently accelerates postoperative recovery, shortens hospital stay, reduces the total medical costs of the patients and thus is more acceptable than conventional management.

## Background

Lung cancer is a leading cause of death in many developed countries, and surgical resection plays an important role in its treatment. Advances in CT imaging technology have greatly increased the early detection rate of lung cancers, which provides more therapeutic options for patients. However, some patients with central lung cancers have to receive pneumonectomy, a difficult surgical procedure with a high rate of postoperative complications (17–47%) [1–5]. Pneumonectomy reduces the lung capacity by at least 45% and decreases functional lung reserves over a short period of time. More importantly, pneumonectomy increases the incidence of arrhythmia, acute pulmonary edema, and acute respiratory failure, thereby increasing hospital stays and medical costs [6]. With advancement in biomedical technology, patients have become increasingly concerned with health care quality and efficacy. Thus, in modern lung cancer surgery, it is important to optimize therapies of patients with major complications [7]. To reduce the incidence of postoperative complications and increase health care efficacy, multiple therapeutic strategies and perioperative managements have been introduced into the surgical field, including infection controls, nutritional support, improvement of fluid management, and pursuing ideal preoperative evaluation. Among them, a set of perioperative management, named fast-track surgery (FTS), is considered as an optimal approach in general. The FTS, initially proposed by Kehlet, is a combination of various biomedical techniques of perioperative cares for patients undergoing elective operations [8]. The methods adopted in FTS have been shown to effectively reduce common complications and generalized pain of patients. Thus, FTS should also benefit patients of pneumonectomy, which remains to be evaluated, particularly when some points of the original fast-track program such as early exercise and epidural analgesia in fact are current standard practice in most of thoracic surgical departments. To evaluate the beneficial potential of FTS in pneumonectomy, we compared the outcomes of pneumonectomy in the patients of non-small cell lung cancer (NSCLC) who had been treated with FTS to those who received conservative perioperational preparations. Our results indicate that FTS can efficiently accelerate postoperative recovery, shorten hospital stay, and reduce the total medical costs for NSCLC patients. Thus, this study indicates that FTS remains feasible and useful in the setting of NSCLC pneumonectomy.

## Methods

The study was performed in the Department of Thoracic Surgery of the Fourth Affiliated Hospital, Harbin Medical University between June 2012 and March 2014.

### Inclusion criteria

Inpatients were recruited according to the lately developed criteria: (1) diagnosed with primary pulmonary adenocarcinoma or squamous cell carcinoma via biopsies guided by videobronchoscopy or CT scan and never received chemotherapy or radiotherapy; (2) presented with central lung cancers whose chest CT scans strongly indicated the necessity of a pneumonectomy and showed no signs of remote metastases in isotope bone scan, brain MRI, and abdominal CT; (3) did not present with a history of arrhythmias or myocardial infarction; (4) did not present signs of chronic obstructive pulmonary disease (COPD) with a forced expiratory volume in 1 s (FEVl) >2.5 L; (5) did not have interstitial lung disease or asthma preoperatively; (6) were 44–65 years old; and (7) had no hypertension, diabetes, renal dysfunction, or gastrointestinal diseases. Patients should also (1) tolerate preoperative enteral nutrition and thoracic epidural anesthesia without coagulation dysfunction, (2) have a body mass index (BMI) of 18.5–30.0 kg/m^2^, and (3) have a physical status between I and II sets by the American Society of Anesthesiologists (ASA).

### Exclusion criteria

Patients who had neuromuscular diseases and could not receive postoperative chest physiotherapy or had ever received thoracic surgeries were excluded.

### Study endpoints

Major endpoints included surgical complications, such as arrhythmias, pulmonary infection, acute pulmonary edema, stress-induced gastrointestinal bleeding, acute respiratory failure, and urinary tract infections. Secondary endpoints included postoperative body temperature, time to first postoperative flatus, time to defecation, length of hospital stay, and medical costs. In addition, C-reactive protein (CRP) was detected preoperatively and on postoperative day (POD) 1, 3, and 7.

### FTS procedures

#### Randomization

Computer-generated block randomization was initiated by a data manager in the respiratory research group and placed in individual sealed envelopes. Both the surgeon and the thoracic research assistant interviewing potential candidates for the study were blind to the randomization code. Each envelope was opened in front of the patient upon entry into the study after written informed consent was received. Thirty-five patients met criteria stated above were included in this study and randomly assigned to the conservative group and FTS group; they were treated separately as shown in Table 1. In evaluation of the outcomes of patients with different treatments, a thoracic research assistant not knowing the procedures for individual patients was assigned to ensure double blind and minimize potential bias problem due to non-blinding.

**Table 1:** Comparison of perioperative measures between conservative and the FTS groups

Measures	Control group (*n* = 18)	FTS group (*n* = 17)
Preoperative education	Concept of standard manner	Concept of FTS
Preoperative diet	Fasting for 6 h	Took 1000 ml of 10% glucose orally at the night before operation; took 200 ml of 10% glucose orally 2 h before operation
Preoperative sedation to improve sleep	Yes	Yes
Indwelling catheter after anesthesia	Yes	Yes
Intraoperative warming	No	Yes
Postoperative analgesia	Patient-controlled epidural analgesia	Patient-controlled epidural analgesia—oral use of nonsteroidal analgesic painkillers for 48 h
Postoperative amount of fluid	Total postoperative intravenous infusion volume within 24 h should be <1500 ml, with a intravenous infusion rate of 20–30 ml min; if hypotension appeared or urine volume was <20 ml/h, vasoconstrictors were used	Fast intravenous infusion of 250 ml saline within 1 h: the rest were the same as the conservative group
Diet 6 h postoperatively	A small amount of water	400 ml liquid food
Measures to promote bowel movements	None	Chewing gum
Early extubation of urinary catheter	24 h postoperatively	12 h postoperatively
Early exercise	Following patients’ will	Active bed activities of the lower limbs

#### Perioperative management

Patients from the control group were treated conservatively, while patients from the FTS groups were treated by accelerated measures (Table 1).

#### Intraoperative management

An experienced surgeon performed all the major procedures, and operations were performed in the morning. All patients received general anesthesia via double-lumen endotracheal tubes and thoracic epidural anesthesia. The healthy lobes of the lung were ventilated. A smallest incision was cut when possible, and wound was closed using absorbable sutures. All operations were performed via a standard open posterolateral thoracotomy for both the FTS and the conservative groups. Adhesions among the lung, pleura, and diaphragm were dissected. After confirming the location and characteristics of the lesion, pneumonectomy was performed. A bronchial closure device was used to close the main bronchus, the mediastinal lymph nodes were dissected, and, then, an indwelling thoracic closed drainage tube was inserted. The device was continuously closed postoperatively unless it was necessary to adjust the pressure. The chest tube was removed 24 h postoperatively, and the volume and rate of transfusion (≤1500 ml/day) was conservatively controlled to maintain hemodynamic stability.

### Criteria for patient discharge and follow-up

Patients were discharged if the following criteria were met. (1) The thoracic drainage tube was removed, and the patient was encouraged to ambulate freely. (2) The patient displayed proper breathing activities (lack of shortness of breath, wheezing, or crackles, oxygen saturation >94%) without any difficulties. (3) There were no severe complications on discharging the patient, and complications were effectively controlled. Follow-up was provided up to POD 30.

### Calculation of medical costs

We recorded and calculated the total medical costs through hospital computational system based on daily report of nurse station. This system automatically summarized all costs of medical services including consumption of the operation room, fee for surgeons and anesthetist, perioperative care, daily drug usage, laboratory tests, physical checkups, and rehabilitation.

### Statistical analysis

The SPSS 13.0 statistical software program was used for all statistic analyses. Data were expressed as mean ± standard deviation. Comparisons between two groups were performed using Student’s *t* test and between three groups using one-way repeat measurement ANOVA. *P* < 0.05 was considered statistically significant.

## Results

### General characteristics of the patients

**Fig. 1:**
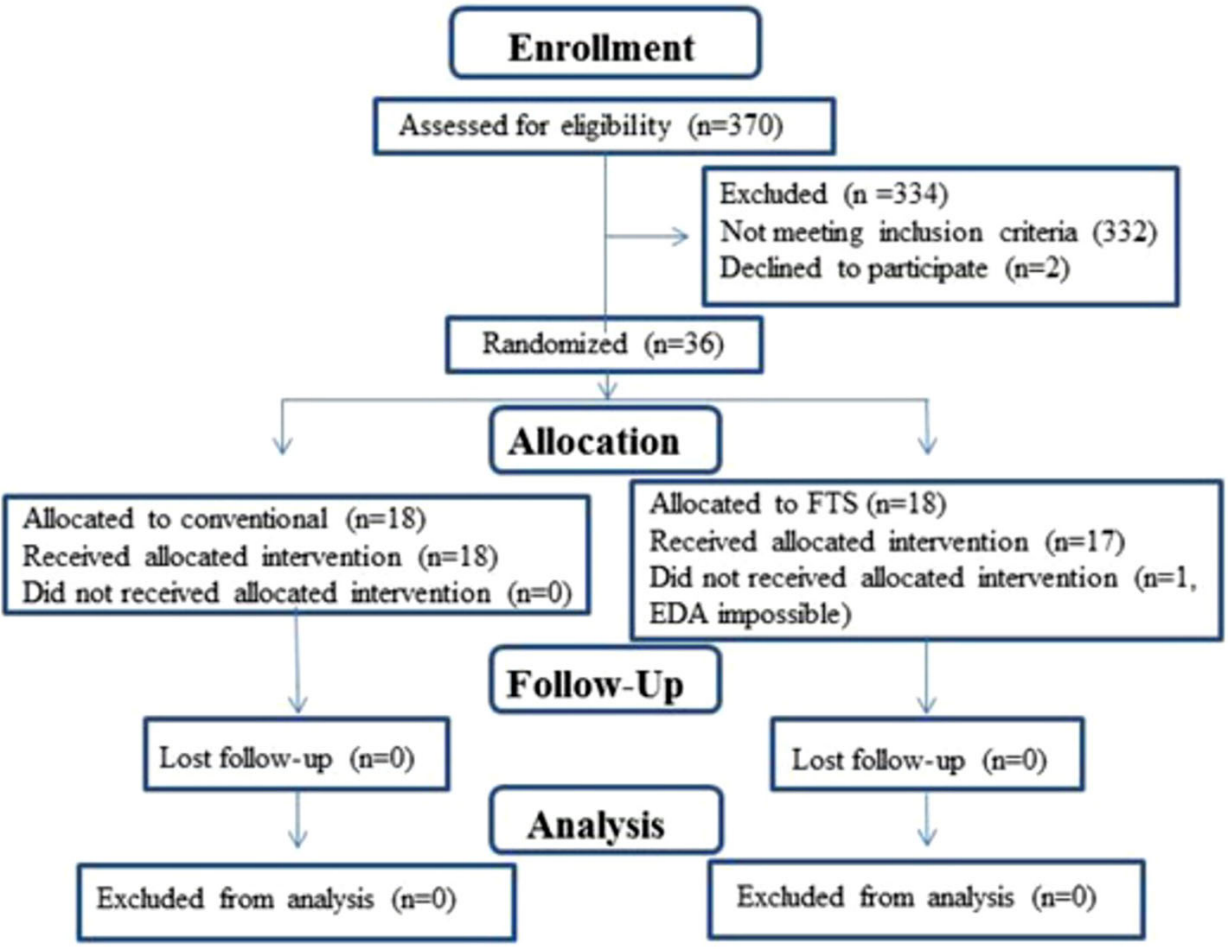
CONSORT flow diagram showing critical reporting features for our randomized clinical trial and its outcomes. *Abbreviation*: *EDA* epidural anesthesia

Among 370 patients of lung cancer resections, 36 fell in our inclusion criteria for pneumonectomy and assigned randomly into the control and FTS groups. As shown in the CONSORT flow diagram (Fig. 1), all patients (*n* = 36) except one in the FTS group went through all the designed procedures. The two groups were comparable in the age, gender, BMI, operative side (left or right), ASA score, and surgical procedures. From these patients in both groups, T2 stages were predominant (Table 2). No death was recorded and no blood transfusion was given to any of the patients. Tracheal intubation was removed postoperatively in the operating room when patients could spontaneously breath and cough strongly, swallowing functioned well, and blood gas analysis results were normal without laryngeal obstruction. All of the patients were sent to the care unit of the general ward and no patient required admitting to the intensive care unit for mechanical ventilation. Complete resection, classified as R0, was defined as pathologic evidence of negative tissue margins and determined by the surgeon, in which all detectable lesions had been removed. Postoperative complications of patients were alleviated and cured after treatments before being discharged.

**Table 2:** Overview of patient characteristics in the FTS and the conservative groups

Group	Control (*n* = 18)	FTS (*n* = 17)	*P* value
Age (year)	56.6 (50–65)	55.1 (44–65)	0.931
Sex			1.000
Male	14 (78%)	13 (76%)	
Female	4 (22%)	4 (24%)	
ASA score			0.877
ASA 1	10 (56%)	9 (53%)	
ASA 2	8 (44%)	8 (47%)	
BMI (kg/m^2^)	25.6 (18.7–29.8)	26.8 (18.5–30.0)	0.832
FEV1 (l)	2.8 (2.5–3.7)	2.9 (2.5–3.9)	1.000
*T* stage			0.903
T2	12 (67%)	11 (65%)	
T3	6 (33%)	6 (35%)	
Operation side			0.658
Left	14 (78%)	15 (88%)	
Right	4 (22%)	2 (12%)	
Pathology			1.000
Squamous carcinoma	Cell 16 (89%)	15 (88%)	
Adenocarcinoma	2 (11%)	2 (12%)	

*FEV1* forced expiratory volume in 1 s, *ASA* American Society of Anesthesiologists, *BMI* body mass index

### Indices of clinical observation

Compared to the control group, the FTS group showed significantly (*P* < 0.05) shorter time to the first postoperative flatus, the time of defecation, and the length of hospital stay. Correspondingly, total medical costs were lowered significantly (Table 3). However, no significant difference was found in the postoperative body temperatures (*P* = 0.110). The postoperative complications occurred in 23.5% of the 17 patients in the FTS group (one case each of arrhythmia, pulmonary infection, acute pulmonary edema, and acute respiratory failure) and in 33.3% of the 18 patients in the control group (one case each of arrhythmia, pulmonary infection, acute pulmonary edema, stress-induced gastrointestinal bleeding, acute respiratory failure, and urinary tract infection). The difference in the incidence of complications between the two groups was insignificant. Additionally, CRP levels throughout the POD were significantly higher than those found preoperatively in both groups (all *P* < 0.05, see Table 4), which started on POD 1 with peak on POD 7. Follow-ups on POD 30 showed that the conditions of all these patients were stable. No patient was readmitted since discharged in both groups.

**Table 3:** Indices of clinical observations of the two groups

Indices of clinical observation	Control group	FTS group	*P* value
	(*n* = 18)	(*n* = 17)	
Postoperative body temperature (°C)	35.6 ± 0.4	35.8 ± 0.4	0.110
Time to first postoperative flatus (day)	3.1 ± 0.8	1.5 ± 0.6	0.0001
Time to defecation (day)	5.5 ± 1.3	3.4 ± 0.7	0.0001
Length of hospital stay (day)	27.4 ± 6.6	18.1 ± 1.4	0.0001
Total medical cost (RMB, thousand Yuan)	37.2 ± 3.6	29.9 ± 2.7	0.0001
Complications (number of cases)	6	4	0.711
Arrhythmia (cases)	1	1	
Pulmonary infection (cases)	1	1	
Acute pulmonary edema (cases)	1	1	
Stress-induced gastrointestinal bleeding (cases)	1	0	
Acute respiratory failure (cases)	1	1	
Urinary tract infection (cases)	1	0	

Variables were expressed as the mean ± SD

**Table 4:** Perioperative C-reactive protein levels of the two groups (mg/l)

Group	Control group (*n* = 18)	FTS group (*n* = 17)	*P* value
Preoperative	4.08 ± 0.52	4.02 ± 0.47	0.667
POD 1	95.21 ± 13.02	85.64 ± 11.41	0.029
POD 3	167.36 ± 15.86	146.36 ± 15.76	0.0001
POD 7	80.71 ± 8.32	71.36 ± 5.48	0.0001

Variables were expressed as the mean ± SD

*POD* postoperative day

## Discussion

As an optimized surgical approach, FTS is a combination of several beneficial perioperative measures to avoid general morbidity and high complication rates while increasing the speed of postoperative recovery [9]. The key advantages of FTS are (1) patients are well informed and thoroughly evaluated preoperatively, making patients well prepared both mentally and physically for their forthcoming procedures, and (2) FTS can reduce stress surrounding perioperative period. Reducing postoperative stress that might be experienced by the patient decreases many adverse reactions and accelerates recovery [10–19]. As a result, the FTS shortens the recovery time, reduces postoperative complications, lowers the mortality rate, decreases the length of hospital stay, and improves many other perioperative criteria in patients.

Pneumonectomy is a high-risk operation, and we set seven quite strict inclusion criteria for the trial including relatively young age interval between 44 and 65 years and no obvious comorbidity. In this study, both groups were comparable in gender, age, operative site, *T* stage, preoperative FEV1, ASA score, BMI, pathological stage, and surgical procedures performed, which were selected from 370 cases of lung cancer resections with clear records including lobectomies, wedge resection, and anatomical segmentectomies. In addition, we also took double-blind method to avoid sampling bias.

In the preoperative period, patients in both groups were educated for the procedures of postoperative care and the use of chest tube. We also included some contents based on previous studies and theoretical considerations [20, 21]. For instance, an educational plan of postoperative care can reduce anxiety and enhance postsurgical recovery. We have also learned that if physicians and nurses co-operatively educate patients conventional behaviors such as taking exercise and quitting smoking, the rate of perioperative complications could be reduced [22]. Normally, it would take 6 h for gastric emptying of solid food and 2 h for liquids [23]. In FTS, preoperative fasting for 2 h could alleviate the decreased insulin sensitivity but did not increase the risks of complications such as intraoperative regurgitation or aspiration. Proper preoperative glucose load can promote early phase insulin secretion, which is an important measure for improving insulin resistance [24]. These special measures included orally taking 1000 ml of 10% glucose at the night prior to surgery and then orally taking 200 ml of 10% glucose 2 h before surgery, improving sleep and keeping the urinary catheter after general anesthesia. Similar to the techniques of traditional thoracotomic pneumonectomy, surgical incision was also designed to be as small as possible.

During surgery, surgeons should cooperate with rotating nurses and anesthesiologists with particular attention to heating and washing the patients with warm water, and in observing conservative control of general anesthesia, epidural anesthesia, and fluid management, thereby reducing or blocking any potential stress. Another routine measure of surgical procedures in our department was to avoid heat loss in patients by using air heaters on the operative table and administrating warm intravenous fluids. In our FTS protocol, we also considered to warm the operating room to 24 °C to avoid potential hypothermia in our patients. This could contribute to the stability of patients’ conditions although it was reported that heating the operating room did not result in any significant improvement of core body temperature (35.8 versus 35.6 °C) [25]. In addition, efforts were paid for establishing optimal analgesia levels, which can contribute to the recovery of body functions and reduce the risk for dyspnea induced by lying in bed [26, 27].

After surgery, patients were secured satisfactory analgesia using patient-controlled epidural analgesia and oral use of nonsteroidal analgesic painkillers, which helped to eliminate the possibilities of postoperative gastrointestinal dysfunction (e.g., nausea and vomiting) caused by opioid drugs. As a result, our FTS in the NSCLC pneumonectomy did yield the effects of accelerating postoperative recovery, shortening hospital stay, and reducing the total medical costs. The recovery was indicated by the earlier occurrence of the first postoperative flatus and time to defecation in the FTS group. Following pneumonectomy, hemodynamic, renal and respiratory issues are the major traditional postoperative concerns. However, the first postoperative flatus is an indication of postoperative recovery of gastrointestinal functions and the result of overall recovery. This result is consistent with that the shorter the time is the better the intra-gastrointestinal nutritional support and overall functional recovery is obtained [17]. Thus, we used this parameter while monitoring other indices.

Another worth noting issue is the fluid and nutrient managements. It is known that conservative fluid management may result in postoperative hypovolemia and loss of perfusion to the tip of the microvillus, triggering apoptosis and potentially necrosis, which typically requires about 3 days of recovery [28]. Postoperatively fast intravenous infusion small amount of fluids in the FTS group within 1 h could alleviate the occurrence of such injuries. For the nutrition, allowing eating/drinking until late in the day before surgery and restarting the feeding soon after the procedure has many advantages. As shown in this study, early eating/drinking promoted bowel movements. Additionally, proper postoperative exercise might shorten the time to the first postoperative flatus and time to defecation. Intensive motor activity has long been known to be a crucial factor to prevent postoperative complications. Prolonged bed rest is associated with impaired pulmonary function, increased muscle loss, delayed motility of the intestines, and increased risk of thromboembolism. However, no disadvantages have been described due to early and intensive activity. Patients undergoing pneumonectomy in this study were encouraged to adopt early postoperative active bed activities of the lower limbs.

Consistent with the general conditions at different stages of the FTS, CRP levels also changed markedly. CRP is known as an acute-phase protein of hepatic origin, the levels of which rose in response to inflammation following interleukin-6 secretion from macrophages and T cells, reflecting the intensities of stress [29]. CRP levels of patients in both groups significantly increased due to operative stress. That the CRP levels of patients in the FTS group were significantly lower than that in the control group on POD 1, 3, 7 indicates a relatively milder stress, which is consistent with the fast recovery following the pneumonectomy in FTS group.

As for the primary endpoints of our study, we also observed and analyzed postoperative complications including arrhythmia, pulmonary infection, acute pulmonary edema, stress-induced gastrointestinal bleeding, acute respiratory failure, and urinary tract infection. We found that patients in the FTS group had a relatively low incidence of the complications although we failed to obtain statistically significant difference. This resembled the observations made by Ichiki and colleagues [18] but was lower than that reported by Shapiro et al. [30]. Our finding was likely associated with the general improvement of surgical conditions. In fact, some procedures of the fast-track program, such as early exercise and epidural analgesia, are current standard practice in most of thoracic surgical departments; improvements in general conditions of anesthesia, analgesia, ward care, nutrition, and physical activity have greatly improved patients’ conditions [18, 19, 25]. These measures likely make the differences in the complications between the two groups less than originally expected, along with the shorter intensive care and hospital stays. Nevertheless, the finding that the FTS group had a shorter hospital stay and lower medical costs as compared with the conservative group itself is a strong sign that the patients in the FTS group had a better outcome than the ones received conservative treatment.

Noteworthy is that the length of hospital stay was relatively long compared with medical centers in Western countries if not considering the differences in the specific disease conditions and in cultural backgrounds. Relative to the FTS in non-thoracic diseases, pneumonectomy itself requires longer hospital stay. It is a common sense that any surgical program that is designed to shorten hospital length of stay is at risk for discharging patients. In Chinese medical centers, length of hospital stay includes not only the essential times of preparation, operation, and postoperative monitoring in intensive care unit but also major part of the rehabilitation. Importantly, the duration of hospitalization in this FTS was shorter than that of similar studies, even less than those known to have less postoperative complications than that of the present study [31]. As a result, the readmission rate in this study was generally lower, which supports adoption of the FTS in NSCLC pneumonectomy.

## Conclusions

FTS included patient-controlled epidural anesthesia, conservative intraoperative fluid administration, immediate postoperative extubation, early mobilization, and discharge goals. Applying FTS in NSCLC pneumonectomy can effectively alleviate postoperative stress, shorten the length of hospital stay, and improve the health of the patient. Due to the small sample size, excellent preoperative status, and improved conservative cares in our recruited patients, this study might not reflect FTS effects on postoperative complications sufficiently. In addition, the postoperative follow-ups were relatively short and might not fully reflect the prognosis. Thus, a future research study is required to assess the safety and long-term effects of the FTS pathway on NSCLC pneumonectomy.

## Abbreviations

ASA: American Society of Anesthesiologists
BMI: Body mass index
COPD: Chronic obstructive pulmonary disease
CRP: C-reactive protein
FEVl: Forced expiratory volume in 1 s
FTS: Fast-track surgery
NSCLC: Non-small cell lung cancer
POD: Postoperative day
